# Inflammation After Transcatheter Aortic Valve Implantation: Mechanisms, Risk Stratification, and Therapeutic Implications

**DOI:** 10.31083/RCM52850

**Published:** 2026-09-17

**Authors:** Mihail Celeski, Michele Marchetta, Emanuele Sammartini, Antonio Sorropago, Mattia Basile, Matteo Morello, Carmine Salzillo, Marco Bernardi, Pasquale Mollo, Francisco José Romeo, Arturo Cesaro, Michele Golino

**Affiliations:** ^1^Department of Cardiology, Fabrizio Spaziani Hospital, 03100 Frosinone, Italy; ^2^Berne Cardiovascular Research Center and Division of Cardiology, University of Virginia, Charlottesville, VA 22903, USA; ^3^Department of Medicine, Vanderbilt University Medical Center, Nashville, TN 37232, USA; ^4^Department of Cardiology, Policlinico Casilino, 00169 Rome, Italy; ^5^Department of Experimental and Clinical Medicine, Magna Graecia University, 88100 Catanzaro, Italy; ^6^Department of Interventional Cardiology, Policlinico di Monza, 20900 Monza, Italy; ^7^Cardiology Department, La Paz University Hospital, 28046 Madrid, Spain; ^8^Fundación de Investigación IDIPAZ, 28029 Madrid, Spain; ^9^Department of Molecular and Translational Medicine, University of Brescia, 25121 Brescia, Italy; ^10^Department of Cardiovascular and Pulmonary Sciences, Università Cattolica del Sacro Cuore, 00168 Rome, Italy; ^11^Department of Cardiology, Santa Maria Goretti, 04100 Latina, Italy; ^12^Department of Cardiology, University of Miami Miller School of Medicine, Jackson Memorial Hospital, Miami, FL 33136, USA; ^13^Department of Translational Medical Sciences, University of Campania Luigi Vanvitelli, 80138 Naples, Italy; ^14^Division of Cardiology, A.O.R.N. “Sant'Anna e San Sebastiano”, 81100 Caserta, Italy; ^15^Pauley Heart Center, Division of Cardiology, Virginia Commonwealth University, Richmond, VA 23284, USA; ^16^Division of Cardiology, University of Florida College of Medicine, Jacksonville, FL 32209, USA

**Keywords:** aortic stenosis, transcatheter aortic valve implantation, inflammation, anti-inflammatory therapy, phenotyping

## Abstract

Transcatheter aortic valve implantation (TAVI) elicits a sterile inflammatory response that is almost universal, yet highly heterogeneous in both magnitude and clinical impact. Initiated by endothelial injury, ischemia–reperfusion, and residual bioprosthetic immunogenicity, peri-procedural inflammation converges on activation of the NOD-like receptor family pyrin domain-containing 3 (NLRP3) inflammasome and downstream interleukin-1β (IL-1β) and IL-6 signaling, leading to increased C-reactive protein levels. This response is further modulated by patient susceptibility, procedural complexity, and device-specific characteristics. While a controlled response promotes tissue repair and healing, exaggerated activation, which manifests as systemic inflammatory response syndrome in up to two-thirds of patients, has been consistently associated with higher mortality, conduction disturbances, new-onset atrial fibrillation, acute kidney injury (AKI), and subclinical leaflet thrombosis. Emerging evidence also implicates gut microbiome-derived metabolites and xenoantigen-driven adaptive immunity as additional contributors to the overall inflammatory burden. Serial biomarker assessment, including C-reactive protein trajectories, neutrophil-to-lymphocyte ratio, cancer antigen 125 (CA-125), and novel composite indices such as the neutrophil percentage-to-albumin ratio, combined with multimodality imaging, allows increasingly refined risk stratification. However, no anti-inflammatory therapy is currently established for routine use after TAVI. Colchicine has shown proof-of-concept signals, sodium–glucose cotransporter 2 inhibitors have demonstrated clinical benefit with pleiotropic anti-inflammatory properties, and lessons from IL-1 and IL-6 inhibition trials in ischemic heart disease provide a translational roadmap. This narrative review synthesizes the epidemiology, mechanisms, clinical consequences, and assessment of peri-TAVI inflammation. Moreover, this review proposes a pragmatic inflammatory subphenotyping framework that integrates baseline risk, procedural exposure, and biomarker kinetics. Such a framework may help bridge the gap between observational associations and precision-based therapeutic strategies, shifting peri-TAVI inflammation from a prognostic marker to a modifiable determinant of outcome.

## 1. Introduction

Transcatheter aortic valve implantation (TAVI) is increasingly used in younger and lower-risk populations, reflecting advances in device technology and procedural safety [1,2]. However, despite these encouraging trends, TAVI carries potential complications, including vascular injury, conduction disturbances, and thromboembolic events. In this context, growing evidence links inflammation to several adverse post-procedural outcomes [3,4].

Indeed, TAVI triggers a complex inflammatory response: indispensable, yet overt activation is associated with adverse clinical outcomes. Procedural factors—endothelial disruption, exposure to prosthetic material, and transient hypoperfusion—rapidly activate innate immune pathways and cytokine release after valve implantation [3,4]. This initial adaptive response promotes endothelial healing and remodeling; however, it might become exaggerated and dysregulated in a subset of patients, leading to systemic inflammatory response syndrome (SIRS), expressed as leukocytosis, fever, and hemodynamic instability [3,5]. SIRS has been consistently associated with increased short- and long-term mortality, highlighting the clinical significance of post-TAVI inflammatory burden [3].

Peri-procedural inflammation after TAVI is increasingly recognized as an active contributor to post-procedural complications rather than a nonspecific consequence of procedural stress. Mechanistic and clinical data implicate the interleukin 1β (IL-1β)–interleukin 6 (IL-6)–C-reactive protein (CRP) axis as a central pathway linking innate immune activation to systemic inflammation and downstream adverse events [6,7]. This aligns with broader cardiovascular research showing that targeted anti-inflammatory therapies can reduce cardiovascular risk [8,9]. Notwithstanding the highly heterogeneous mechanisms underlying the post-TAVI inflammatory response [10,11,12], inflammatory biomarkers, such as CRP, leukocyte count, and circulating cytokines, may help identify patients at higher risk of adverse outcomes [4,13].

Understanding peri-procedural inflammation may improve risk stratification and patient management after TAVI. This narrative review summarizes its epidemiology, mechanisms, and clinical implications, and examines its transition from prognostic marker to potential therapeutic target. We also propose an inflammatory subphenotyping framework to support tailored anti-inflammatory strategies in TAVI.

## 2. Epidemiology

Inflammation is nearly universal after TAVI, with most patients showing measurable increases in inflammatory biomarkers during the early post-procedural period. Observational data suggest that approximately two-thirds of patients develop significant biomarker elevation within the first 5 days, particularly during the first 72 hours [10].

The clinical spectrum ranges from isolated laboratory abnormalities to overt SIRS. Importantly, post-TAVI inflammation is predominantly sterile, with confirmed infection reported in only 2.6% of patients in a cohort of more than 1200, despite marked inflammatory abnormalities [13]. Misinterpretation of this response as an infection may contribute to unnecessary antibiotic use. Using the classic SIRS criteria, up to 63% of patients develop SIRS after TAVI [3,4,5,14,15,16,17]. Higher rates have been reported when more sensitive biomarker-based or early hemodynamic definitions are applied, particularly in high-risk cohorts and in transapical procedures [14,15,17]. The prognostic impact of SIRS after TAVI is substantial but nuanced. Reported 30-day mortality rates in patients meeting ≥2 SIRS criteria range from 5.3% to 22%, and 1-year mortality from 21% to 53% [4]. However, one study found no association between post-TAVI SIRS (≥2 criteria) and 2-year mortality, and only severe SIRS (meeting all 4 criteria) independently predicted 6-month mortality [5,18]. Elevated baseline CRP levels also predict mortality after TAVI, with a threshold of >0.10 mg/dL (>1.0 mg/L) predicting all-cause death within 3 months (hazard ratio (HR) 2.78, 95% CI 1.30–5.95) and ≥0.20 mg/dL (≥2.0 mg/L) predicting 2-year mortality (HR 2.42) [19,20]. These findings underscore that both the severity and persistence of the inflammatory response, rather than its mere presence, determine clinical impact.

## 3. Potential Mechanisms of TAVI-Induced Inflammation

TAVI-induced inflammation results from the convergence of multiple mechanisms, including endothelial injury, ischemia-reperfusion, and biomaterial-induced immune activation, all leading to innate immune signaling and cytokine release [4].

Endothelial injury is the key initiating event. Mechanical valve deployment within a heavily calcified annulus disrupts the endothelium, exposing the subendothelial matrix, and activating coagulation and inflammatory pathways. This response is amplified by complement activation, cytokine release, and local hypoxia, promoting a prothrombotic phenotype [21] and contributing to the thrombo-inflammatory activation post-TAVI [3]. Endothelial activation triggers NF-κB–mediated cytokine production (e.g., IL-1, IL-6, IL-8) while complement and coagulation pathways interact bidirectionally to sustain inflammation [21,22]. Increased CD144-positive endothelial microparticles have been observed in patients developing SIRS after TAVI [16], reflecting endothelial dysfunction and thrombo-inflammatory activation as suggested by data from aortic stenosis and TAVI [23].

Ischemia-reperfusion injury (IRI) further contributes. Rapid ventricular pacing and peri-procedural severe aortic regurgitation induce transient hypoperfusion during valve implantation, followed by reperfusion-driven reactive oxygen species (ROS) generation and release of damage-associated molecular patterns (DAMPs) [4]. These activate pattern recognition receptors—such as Toll-like and NOD-like receptors [3,7,24]—and downstream inflammatory signaling, particularly through NF-κB [3,7,24].

Hypoperfusion may also impair intestinal barrier integrity, allowing translocation of bacterial endotoxins, such as lipopolysaccharides, into the bloodstream, further enhancing the downstream inflammatory signaling pathways and host immune response [4,25].

Beyond acute endotoxin translocation, emerging evidence suggests that the intestinal microbiota may actively modulate the systemic inflammatory response in aortic valve disease. Trimethylamine N-oxide (TMAO), a metabolite produced from dietary components by gut bacteria, promotes fibrotic activation of quiescent valvular interstitial cells via endoplasmic reticulum stress and aggravates inflammation in human aortic valve interstitial cells by regulating macrophage M1 polarization through a methyltransferase-like 3 (Mettl3)-mediated N6-methyladenosine (m6A)/YTH N6-methyladenosine RNA binding protein F2 (YTHDF2) pathway [26,27]. *In vivo*, dietary choline supplementation increases TMAO levels and aortic valve fibrosis, effects that are reversed by inhibition of trimethylamine formation [28]. A scoping review confirmed that TMAO is the most consistently identified gut-derived metabolite linked to calcific aortic valve disease, with characteristic microbiome compositions observed in affected patients [29]. The role of the gut microbiota-TMAO axis in the context of TAVI remains at a preclinical and hypothesis-generating stage. Although experimental and observational studies support a role for microbiome-derived metabolites such as TMAO in valvular inflammation and calcific aortic valve disease, no direct evidence currently links these pathways to post-TAVI inflammatory burden or clinical outcomes. Whether specific gut microbiome profiles predispose to exaggerated inflammatory activation after TAVI remains unknown. The mechanistic hypotheses discussed in this section should therefore be interpreted as a biological rationale for future translational research rather than as established contributors to peri-procedural inflammation.

Furthermore, bioprosthetic valve immunogenicity represents an additional pathway. Despite chemical fixation, xenogeneic tissues retain antigenic components triggering innate and adaptive immune responses, complement activation, and thrombo-inflammatory signaling, potentially affecting valve durability [30]. Although glutaraldehyde fixation is used during valve manufacturing to preserve tissue integrity, it may leave residual aldehyde groups that favor calcification and immune activation. Current pretreatment strategies may also fail to completely remove relevant xenoantigens, including α-Gal and N-glycolylneuraminic acid [30], leading to persistent increases in α-Gal–specific immunoglobulin G3 (IgG3) and complement factor complement component 3a (C3a) in post-TAVI patients months after the procedure [31]. These processes converge on activation of the NOD-like receptor family pyrin domain-containing 3 (NLRP3) inflammasome [31,32], amplifying IL-1β- and IL-18-mediated inflammation [7,33]. The magnitude of this response may vary by valve platform, with self-expanding valves associated with a greater inflammatory response than balloon-expandable valves [11]. Novel valve coating strategies are being developed to address bioprosthetic immunogenicity. A recent preclinical study demonstrated that a reactive oxygen species–responsive glycocalyx-mimetic hydrogel coating with integrated anticoagulant and anti-inflammatory properties reduced calcification in animal models, supporting the concept that surface engineering of bioprosthetic valves may mitigate immune-mediated structural degeneration [34].

## 4. Temporal Dynamics of Peri-TAVI Inflammation

An early phase, driven by procedural trauma, vascular manipulation, and ischemia-reperfusion, is characterized by rapid release of pro-inflammatory cytokines, particularly IL-6, IL-8, TNF-α, and IL-1β, reflecting innate immune activation and endothelial stress. Leukocyte count and body temperature typically peak within the first 24 hours, whereas CRP rises more slowly and usually peaks at 48–72 hours as part of the hepatic acute-phase response [3,14]. This temporal dissociation highlights the hierarchical nature of post-TAVI inflammation, in which early local injury signals propagate into a broader systemic response. Clinically, early cytokine activation may identify patients at risk for rapid inflammatory complications, while serial assessment of CRP and other conventional biomarkers, including leukocyte count, neutrophil predominance, and the neutrophil-to-lymphocyte ratio, may better capture the magnitude and persistence of inflammation associated with adverse outcomes [4,35]. This has led to increasing emphasis on serial monitoring, rather than reliance on single-time-point measurements.

## 5. Drivers of Inflammatory Heterogeneity and Subphenotyping After TAVI

Peri-TAVI inflammation is heterogeneous and reflects the interplay of baseline patient characteristics, procedural complexity, and device-specific factors. These determinants influence not only the magnitude of the inflammatory response, but also its temporal profile and clinical consequences. Recognizing this variability is essential for risk stratification and for the development of a clinically meaningful inflammatory subphenotyping framework.

### 5.1 Patient-Related Determinants

Advanced age, chronic comorbidities, and pre-existing systemic inflammation may lower the threshold for exaggerated immune activation after procedural injury. Aging is associated with “inflammaging”, a condition characterized by persistent low-grade innate immune activation, elevated circulating cytokines, and impaired adaptive immune regulation, and both age and body mass index have been identified as predictors of SIRS after TAVI [12]. Frailty and inflammation appear mechanistically linked in TAVI patients. Frail patients demonstrate elevated baseline CRP levels (5.9 vs. 3.7 mg/dL, *p* = 0.001) and significantly higher levels of intermediate monocytes (CD14++CD16+), which are pro-inflammatory cells that secrete IL-1β, IL-6, IL-8, and TNF-α. Both frailty and elevated intermediate monocytes independently predict early mortality after TAVI in multivariate analysis [36].

Certain patient subgroups may be particularly vulnerable to inflammation-mediated adverse outcomes. Among diabetic patients, severe SIRS is more strongly associated with 6-month mortality (HR: 4.12) compared to non-diabetic patients (HR: 1.74, interaction *p* = 0.007). Pre-procedural T lymphocyte profiles also influence outcomes, with low T helper 2 cells (which negatively regulate immune response) and high T helper 17 cells (which secrete IL-17) predicting increased 1-year mortality [4].

In addition, conditions such as chronic obstructive pulmonary disease, atherosclerosis, obesity, and chronic kidney disease contribute to a pro-inflammatory and oxidative milieu that may predispose to exaggerated immune activation after valve implantation [37,38]. These overlapping conditions may amplify the inflammatory response to endothelial injury and ischemia-reperfusion during TAVI, increasing the likelihood of hyperinflammatory trajectories. Conversely, patients with lower comorbidity burden and greater physiological reserve may exhibit a more controlled response, supporting the importance of baseline patient profiling and pre-procedural inflammatory biomarkers for risk stratification. Additionally, the composition of the intestinal microbiota is emerging as a patient-related determinant of inflammatory susceptibility. TMAO has been shown to promote osteogenic differentiation and inflammatory activation in valvular interstitial cells through endoplasmic reticulum and mitochondrial stress pathways, and characteristic gut microbiome profiles have been associated with calcific aortic valve disease [29,39]. Whether specific microbiome compositions predispose to exaggerated post-TAVI inflammatory responses warrants further investigation.

### 5.2 Valve and Procedural Characteristics

Procedural and device-related factors are major determinants of inflammatory heterogeneity after TAVI. Access route is particularly relevant, as more invasive approaches, especially transapical TAVI, have been associated with a higher incidence of SIRS than transfemoral procedures, likely because of greater tissue trauma and myocardial injury [14]. Additional procedural contributors include contrast exposure, anesthetic strategy, repeated rapid pacing, post-dilatation, and vascular complications, all of which may amplify systemic immune activation [3,10,40].

Valve-specific characteristics also influence inflammatory burden. Heavily calcified valves, especially in patients with elevated lipoprotein (a) [41] and unfavorable annular anatomy, may increase endothelial stress and procedural complexity, whereas self-expanding valves appear to elicit a more pronounced inflammatory response than balloon-expandable systems, likely owing to sustained radial force and prolonged interaction with the aortic wall [11,42]. However, available evidence supporting device-specific differences in inflammatory activation is derived predominantly from observational studies. Patients receiving different valve platforms frequently differ with respect to anatomical complexity, calcification burden, implantation characteristics, and procedural strategy, all of which may independently influence inflammatory activation. Consequently, residual confounding and selection bias cannot be excluded. However, the consistent observation across multiple independent cohorts that self-expanding valves are associated with greater inflammatory activation provides a biologically plausible signal, given the higher radial forces and more extensive interaction with the native annular tissue inherent to this platform. These findings should be interpreted as hypothesis-generating and warrant confirmation through prospective studies incorporating standardized inflammatory biomarker protocols across valve types. Furthermore, commissural alignment and newer valve platforms may further modulate inflammation by affecting leaflet stress, flow patterns, and vascular trauma [4,18,43,44]. In addition, microembolization during valve deployment represents an important procedural mechanism linking device manipulation to distal ischemic and inflammatory injury, particularly in the cerebral circulation [45,46,47].

Implantation technique may further modulate procedural trauma and its inflammatory consequences. The Optimize PRO study demonstrated that systematic use of the cusp overlap technique with self-expanding valves, combined with standardized procedural steps, reduced the rate of permanent pacemaker implantation to 5.8% when all four protocol steps were followed (n = 1000), suggesting that optimized deployment strategies may attenuate both mechanical and inflammatory injury [48].

## 6. Clinical Consequences of Peri-TAVI Inflammation

Peri-TAVI inflammation has important prognostic implications across both the early and late phases after the procedure. The development of SIRS, as well as higher baseline levels of inflammatory biomarkers such as high-sensitivity C-reactive protein (hs-CRP) and interferon-gamma (IFN-γ), has been associated with an increased risk of mortality and unplanned hospitalization, supporting the clinical relevance of inflammatory burden before and after TAVI [49].

Beyond direct mechanical injury, peri-annular inflammation may promote conduction disturbances and increase the likelihood of permanent pacemaker implantation, with elevated inflammatory indices such as the neutrophil-to-lymphocyte ratio associated with new conduction abnormalities [50,51]. Systemic and local inflammation have also been implicated in new-onset atrial fibrillation through cytokine-mediated electrophysiologic and autonomic effects, with important consequences for thromboembolic risk, prolonged hospitalization, and early mortality [52,53].

Indeed, mechanistic *in vivo* and *ex vivo* studies demonstrate that pro-inflammatory cytokines—such as tumor necrosis factor-α, IL-1, and IL-6—can acutely downregulate connexin43 expression in cardiomyocytes and macrophages, impairing cell-to-cell coupling and prolonging atrioventricular conduction [54].

Similarly, acute kidney injury (AKI) post-TAVI appears to be partly linked to systemic inflammatory activation, as reflected by associations with leukocytosis, CRP, IL-6, and neutrophil-to-lymphocyte ratio (NLR), and SIRS has emerged as an independent predictor of post-procedural AKI [4,55,56,57,58].

The impact of inflammation may also extend beyond the index hospitalization. Several studies have shown that TAVI-related SIRS is associated with increased 30-day and 1-year mortality [3,14,17,59]. However, the causal relationship between peri-procedural inflammation and adverse outcomes after TAVI remains incompletely established. Most available evidence derives from observational studies reporting associations between inflammatory biomarkers and SIRS with clinical events. Consequently, it remains uncertain whether inflammatory activation directly mediates complications such as conduction disturbances and mortality, or whether it primarily reflects procedural complexity, extent of tissue injury, baseline frailty, and comorbidity burden. These explanations are not mutually exclusive, and inflammation may act as both a biological mediator and as an integrated marker of procedural and patient-related vulnerability. The absence of adequately powered randomized trials demonstrating clinical benefit from targeted anti-inflammatory interventions remains a critical gap in the current evidence base and precludes definitive conclusions regarding causality.

## 7. Biomarkers and Imaging for Inflammatory Risk Stratification

Serial biomarker monitoring represents the most practical approach for assessing inflammatory burden after TAVI. Among available biomarkers, CRP remains the most widely used and clinically accessible marker of systemic inflammation. However, in a peri-TAVI setting, its value lies more in its dynamic trajectory than in an absolute threshold. CRP typically rises within 24 hours and peaks at 48–72 hours, reflecting the magnitude and persistence of the acute-phase response [3,14].

Other routinely available markers, including leukocyte count, neutrophil predominance, and the neutrophil-to-lymphocyte ratio, provide additional insight into innate immune activation and have been more specifically associated with the above-mentioned post-TAVI adverse outcomes [4,55,56,57,58]. IL-6 rises earlier than CRP and may better reflect the proximal inflammatory activation; its clinical use remains limited by reduced availability and lack of standardization. Among composite blood cell–based inflammatory indices, the neutrophil percentage-to-albumin ratio (NPAR) has recently emerged as a particularly powerful predictor. In a multicenter study including 2072 patients (retrospective cohort with prospective validation), post-TAVI NPAR demonstrated superior predictive value for all-cause mortality compared with NLR and platelet-to-lymphocyte ratio (PLR), with dynamic increases from baseline associated with a sharp rise in mortality risk [60]. A multimarker approach may further enhance risk stratification. A study evaluating a panel of 9 pre-TAVI biomarkers—including hs-CRP, IL-6, growth differentiation factor 15 (GDF-15), and N-terminal pro-B-type natriuretic peptide (NT-proBNP)—demonstrated significantly improved predictive accuracy for mortality and treatment futility compared with the Society of Thoracic Surgeons (STS) score alone (area under the receiver operating characteristic curve (AUC) 0.82 vs. 0.69), supporting the integration of inflammatory and cardiac biomarkers into pre-procedural risk assessment [61]. Similarly, the Osaka Prognostic Score, an integrated inflammation–nutrition–immune score based on CRP, albumin, and lymphocyte count, has recently been associated with both in-hospital and one-year mortality after TAVI, with better discriminatory performance than its individual components [62]. Of note, biomarker trajectories are more informative than isolated measurements: transient elevations likely reflect physiological responses, whereas persistent or exaggerated increases seem to identify patients at risk of SIRS, thromboinflammatory complications, prolonged hospitalization, and worse long-term outcomes [4,55,56,57,58,63]. However, standardized thresholds, optimal sampling windows, and reproducible biomarker trajectories remain to be defined before these markers can be incorporated into validated post-TAVI risk stratification models. Inter-study variability in assay methodology, patient selection, and sampling protocols further limits the comparability of existing data and underscores the need for harmonized biomarker assessment frameworks in future TAVI registries and trials. Despite evidence from PROMINENT, REDUCE-IT, and STRENGTH showing the strong prognostic value of CRP—surpassing low-density lipoprotein (LDL) cholesterol in predicting recurrent myocardial infarction, stroke, and cardiovascular death [38,64,65,66,67]—no biomarker-guided therapeutic strategy has yet been validated specifically for TAVI. However, integrating baseline inflammatory status with early post-procedural biomarker kinetics may improve risk stratification, identification of patients at high risk, or selection of more accurate biomarkers in future anti-inflammatory trials.

In this context, CA-125 may provide additional complementary information. Synthesized by mesothelial cells and upregulated by both hydrostatic stress and inflammatory stimuli, CA-125 has been associated with congestion and adverse outcomes in heart failure [68]. In observational TAVI studies, higher pre-procedural CA-125 levels have also been linked to increased mortality and major adverse cardiovascular events (MACE) [69], with possible incremental value in frail patients [70].

Imaging plays a complementary role. Cardiac computed tomography (CT) is currently the gold standard for assessing prosthetic leaflet morphology and motion, enabling detection of abnormalities such as hypoattenuated leaflet thickening (HALT)—wedge-shaped or semilunar opacities at the base of the valve leaflets—and its differentiation from infective endocarditis [15,71,72].

Positron emission tomography (PET) imaging provides additional insights into vascular and valvular inflammation. The development of novel probes targeting specific molecular pathways enables both broad detection of inflammatory activity and greater specificity for distinct cell types or processes. For example, 18F-fluorodeoxyglucose PET can assess acute inflammatory activation, while other tracers capture immediate response to therapy (e.g., endothelial adhesion molecules). Probes like 18F-sodium fluoride or 68Ga–fibroblast activation peptide inhibitor identify longer-term cumulative effects of recent inflammation, targeting microcalcification associated with plaque instability, fibroblast activation, and remodeling processes [73]. Hence, molecular imaging application in post-TAVI patients represents a promising opportunity to identify individuals at higher post-procedural risk [74].

Finally, epicardial adipose tissue attenuation on CT has emerged as a promising marker of inflammation and adverse post-TAVI cardiovascular and cerebrovascular outcomes.

Although these imaging biomarkers are predominantly research tools, they may enhance future risk stratification strategies after TAVI [75].

## 8. Proposed Subphenotyping Framework

We propose a pragmatic inflammatory subphenotyping framework for patients undergoing TAVI (Fig. 1), integrating four sequential domains: baseline characteristics, procedural inflammatory exposure, early biomarker response (24–72 hours), and clinical trajectory. Baseline factors—such as age, comorbidities, renal function, baseline CRP levels, left ventricular function, and aortic valve calcification—define inflammatory susceptibility. Procedural factors, including valve platform, implantation depth, post-dilatation, commissural alignment, procedural duration, and vascular complications, further modulate inflammatory burden. These inputs translate into a post-procedural biomarker profile, ranging from mild activation to exaggerated SIRS characterized by marked elevation in CRP, persistent leukocytosis, and high neutrophil-to-lymphocyte ratio.

**Fig. 1.**
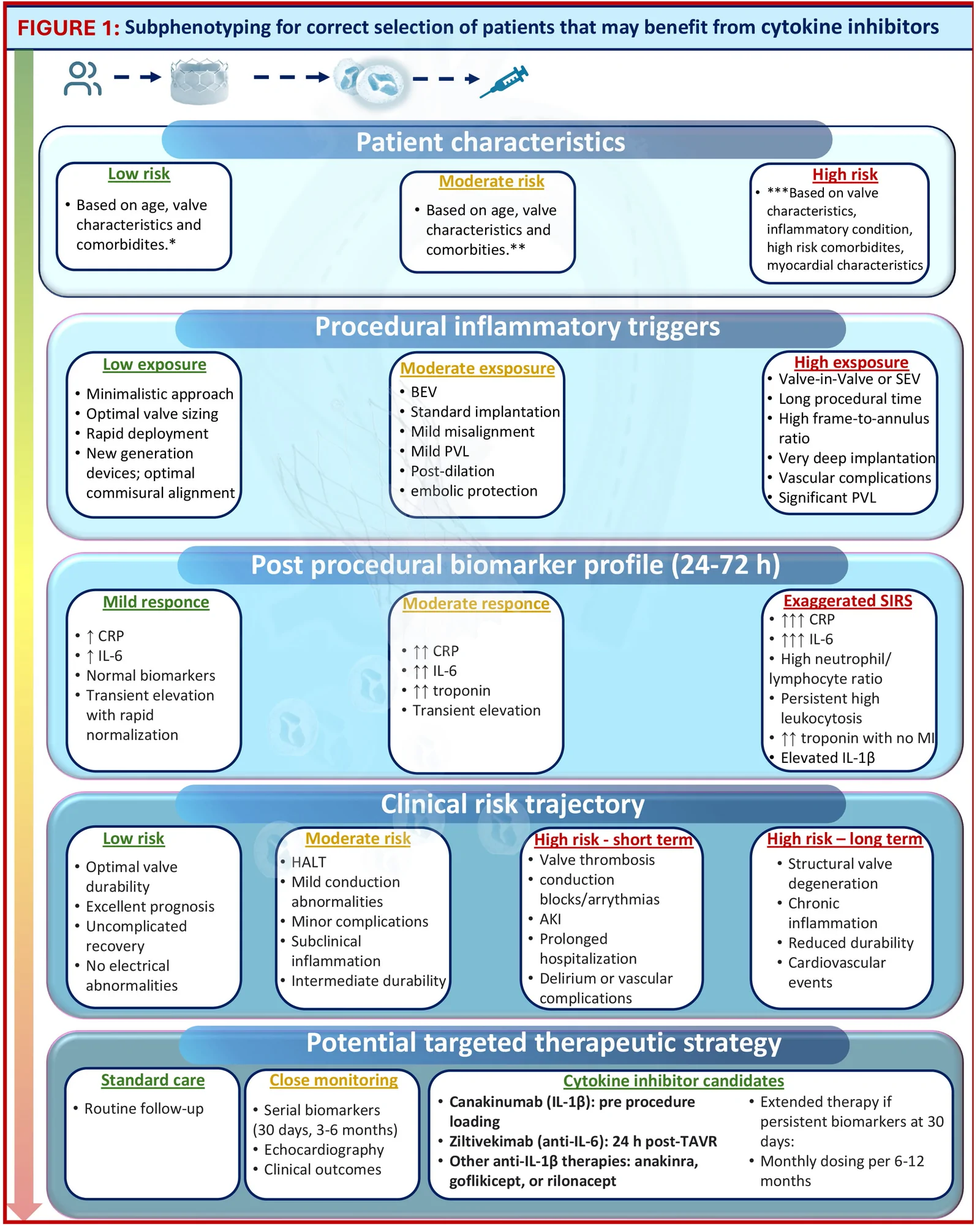
**Subphenotyping for the correct selection of patients that may benefit from cytokine inhibitors**. Schematic framework integrating baseline patient risk, procedural inflammatory exposure, early post-procedural biomarker response (24–72 h), and downstream clinical risk trajectories after transcatheter aortic valve implantation. Patient characteristics and procedural factors determine the magnitude of systemic inflammation, ranging from mild transient biomarker elevation to exaggerated systemic inflammatory response syndrome (SIRS) with marked increases in CRP, IL-6, leukocytosis, and myocardial injury markers. Distinct inflammatory subphenotypes can be associated with short- and long-term outcomes, from uncomplicated recovery and preserved valve durability to valve thrombosis, conduction abnormalities, acute kidney injury, and late structural valve degeneration. Patient characteristics can be divided into 3 categories: low risk, moderate risk, and high risk. *Low risk includes: Young age (<70), no comorbidities, Normal baseline CRP, eGFR >60, preserved LVEF, Low aortic valve calcification. **Moderate risk includes: Diabetes mellitus, high baseline CRP, eGFR 30–60, Prior myocardial infarction, Moderate AV calcification. ***High risk includes: Active inflammatory condition, very high CRP, eGFR <30, Reduced LVEF (<35%), High surgical risk score, Severe AV calcification, Frailty. The proposed stepwise strategy links inflammatory profiling to standard care, intensified monitoring, and selective potential consideration of cytokine-targeted therapies in patients with high inflammatory burden, positioning peri-TAVI inflammation as a modifiable risk factor. Abbreviations: AKI, acute kidney injury; AV, aortic valve; BEV, balloon expandable valve; CRP, C-reactive protein; eGFR, estimated glomerular filtration rate; HALT, hypo-attenuated leaflet thickening; IL, interleukin; LVEF, left ventricular ejection fraction; PVL, paravalvular leak; SEV, self-expandable valve; SIRS, systemic inflammatory response syndrome; TAVI, transcatheter aortic valve implantation.

Hence, this framework links inflammation to outcomes. Patients with low baseline risk, minimal procedural inflammation, and rapidly normalizing biomarkers are more likely to experience an uncomplicated recovery and preserved valve durability. In contrast, those with higher inflammatory risk, greater procedural injury, and exaggerated biomarker responses are at increased risk of conduction abnormalities, arrhythmias, acute kidney injury, leaflet thrombosis, prolonged hospitalization, and structural valve degeneration. Accordingly, this framework supports a shift from reactive toward proactive management by identifying patients who may benefit from closer monitoring and tailored anti-inflammatory strategies. Indeed, inflammatory subphenotyping may allow a precision-based approach in patients undergoing TAVI. However, it should be emphasized that the proposed inflammatory subphenotyping framework is currently conceptual and has not yet undergone prospective validation. Rather than a definitive classification system, it is intended as a hypothesis-generating model designed to integrate clinical, procedural, biomarker, and imaging information into a unified model of inflammatory risk. Future validation could be pursued through prospective multicenter registries incorporating serial inflammatory biomarker assessment, standardized clinical endpoints, and longitudinal imaging follow-up. Notably, existing large-scale TAVI registries and databases with available biomarkers may offer opportunities for preliminary retrospective testing of the proposed classification. External validation and biomarker-guided interventional trials will be necessary before this framework can be considered for routine clinical implementation.

## 9. Current Opportunities for Inflammatory Risk Mitigation After TAVI

At present, no anti-inflammatory strategy has been definitively established for routine use after TAVI.

Therefore, the most actionable approach remains reduction of procedure-related inflammatory triggers together with identification of patients at higher inflammatory risk. In this context, procedural optimization is central and includes preferential use of percutaneous transfemoral access, conscious sedation when feasible, avoidance of prolonged hypotension during valve deployment, minimization of unnecessary pacing or vascular trauma, and careful management of procedural complications. These measures do not target a single cytokine pathway, but may reduce the overall inflammatory burden generated by the intervention. Notably, high-intensity statin therapy—already recommended in most TAVI candidates—exerts pleiotropic anti-inflammatory effects including CRP reduction independent of LDL lowering, and may represent an immediately available, albeit unproven, strategy for peri-procedural inflammatory modulation.

Inflammatory burden after TAVI likely reflects the interaction between procedural injury and baseline patient susceptibility. Biomarker-guided risk stratification—using hs-CRP, IL-6, or neutrophil-to-lymphocyte ratio measured at baseline and in the early post-procedural period—could help identify patients with heightened inflammatory risk who may benefit most from targeted intervention, although optimal markers, thresholds, and timing remain to be defined. Hence, optimization of modifiable contributors to systemic inflammation—such as management of comorbidity burden and broader cardiovascular risk factors—remains reasonable and advisable, particularly in patients with evidence of residual inflammatory risk. Among currently available pharmacologic options, colchicine is the most promising but remains investigational in this setting [76,77]. Its mechanism is partly mediated through inhibition of the NLRP3 inflammasome, a central mediator of sterile inflammation triggered by procedural tissue injury, which provides a plausible mechanistic rationale for its use in the peri-TAVI setting. The Co-STAR trial provides the strongest TAVI-specific randomized evidence for anti-inflammatory treatment to date [77]. In this single-center, double-blind, placebo-controlled trial, 120 patients undergoing transfemoral TAVI were randomized to peri-procedural colchicine or placebo. Colchicine reduced the risk of new-onset atrial fibrillation and permanent pacemaker implantation at 30 days by approximately 2.5-fold compared with placebo. Likewise, it halved the incidence of subclinical leaflet thrombosis on prespecified 4D-CT imaging at 30 days. However, the trial was stopped early due to a higher stroke rate in the colchicine arm and remained underpowered, limiting definitive conclusions. The higher incidence of stroke observed in the colchicine arm deserves careful consideration. While this safety signal contributed to early trial termination, several considerations argue against interpreting it as a definitive causal effect. First, the modest sample size increases the likelihood that the observed difference reflects a chance imbalance rather than a true pharmacological effect. Second, no established biological mechanism supports a prothrombotic or pro-embolic action of colchicine; on the contrary, colchicine has demonstrated favorable cardiovascular safety profiles across multiple large randomized trials in coronary artery disease, including COLCOT [78] and LoDoCo2 [79], with no excess cerebrovascular events. Third, the peri-TAVI setting involves unique stroke risk factors—including catheter manipulation, valve deployment, and calcium embolization—that may act as confounders in a small, prematurely terminated trial. Nonetheless, this finding cannot be dismissed and underscores the need for adequately powered, TAVI-specific trials with pre-specified neurological endpoints and systematic neurocognitive and imaging assessment before colchicine can be considered safe in this population. Accordingly, Co-STAR should be interpreted as proof-of-concept for biomarker- and mechanism-guided anti-inflammatory trials in TAVI rather than support for routine colchicine use. Observational data further support the potential role of colchicine. In a US retrospective cohort analysis of 702 propensity-matched pairs, preoperative colchicine use was associated with a significant reduction in new-onset or worsening atrioventricular block and left bundle-branch block at 1 month (risk ratio (RR) 0.867; 95% CI, 0.756–0.994), although this effect was no longer significant at 6 months.

These findings reinforce the concept of a transient anti-inflammatory benefit and the need for prospective validation [80]. Sodium-glucose cotransporter 2 (SGLT2) inhibitors represent an additional pharmacologic strategy with potential relevance to peri-TAVI inflammatory modulation. Beyond their established hemodynamic effects, SGLT2 inhibitors exert pleiotropic anti-inflammatory properties, including inhibition of the NLRP3 inflammasome via β-hydroxybutyrate-mediated signaling, suppression of NF-κB activation, and reduction of pro-inflammatory cytokines [81,82]. These mechanisms are relevant to the sterile inflammatory cascade triggered by TAVI, which converges on the same NLRP3–IL-1β–IL-6 pathway.

The DapaTAVI trial provides the strongest clinical evidence in this population [83]. In this randomized trial, 1222 patients with severe aortic stenosis undergoing TAVI at high risk for heart failure events were randomized to dapagliflozin 10 mg daily or standard care. At 1 year, dapagliflozin reduced the composite of death or worsening heart failure by 28% (HR 0.72; 95% CI 0.55–0.95; *p* = 0.02), with consistent effects across subgroups. A meta-analysis of 4 studies (12,374 patients) confirmed significant reductions in all-cause mortality and heart failure hospitalization, although largely derived from observational data [84]. Observational data have also suggested a possible association with reduced bioprosthetic valve failure [85].

However, whether these clinical benefits are mediated by anti-inflammatory properties remains uncertain. An exploratory analysis of Dapagliflozin And Prevention of Adverse outcomes in Heart Failure (DAPA-HF) showed no significant reduction in IL-6 or hs-CRP with dapagliflozin at 12 months in heart failure with reduced ejection fraction [86], suggesting that direct cytokine suppression may not be the predominant mechanism. The relative contribution of anti-inflammatory versus hemodynamic effects in the TAVI population warrants dedicated biomarker substudies. Corticosteroids have also been investigated. Two randomized trials in cardiac surgery showed that steroids had no effect on 30-day mortality. In the randomized GLUCO-TAVI trial (n = 100), methylprednisolone one hour before TAVI followed by prednisolone for 5 days was associated with a numerically lower rate of permanent pacemaker implantation at 1 month (8% vs. 16%; RR 0.50; 95% CI 0.16–1.55; *p* = 0.23), although this difference did not reach statistical significance in this pilot study of 100 patients [87]. A meta-analysis of observational studies found no effect of chronic steroids on all-cause death or permanent pacemaker implantation (PPI) after TAVI but an association with major vascular complications [88]. The ongoing EAGLE-TAVI trial (NCT06762145) is comparing intravenous methylprednisolone for 3 days versus placebo, with persistent left bundle-branch block at 30 days as the primary endpoint. Currently, inflammatory risk mitigation after TAVI relies on procedural optimization and biomarker-informed risk stratification (Table 1), highlighting the need for adequately powered, targeted studies in selected high-risk patients.

**Table 1.** **Inflammation-related mechanisms, biomarkers, imaging correlates, clinical consequences, and therapeutic implications in TAVI**.

Domain	Key mechanism	Biomarkers/imaging	Clinical consequences	Mitigation strategy
Endothelial and tissue injury	Mechanical disruption during valve deployment releases DAMPs and activates coagulation-inflammation crosstalk	WBC, hs-CRP, IL-6, endothelial microparticles	SIRS, early hemodynamic instability, prolonged hospitalization	Transfemoral-first strategy, procedural simplification, cusp overlap technique, minimized pacing
Peri-annular conduction injury	Local edema and inflammatory infiltration amplify mechanical trauma to the membranous septum	NLR, CRP	New-onset atrioventricular block, Left bundle branch block, PPI	Baseline conduction profiling; colchicine (proof-of-concept)
Atrial inflammatory remodeling	Cytokine-mediated oxidative stress, autonomic activation, and calcium-dependent electrophysiologic changes	hs-CRP, IL-6, NLR	New-onset atrial fibrillation, thromboembolic risk, ↑ short-term mortality	Identification of high-risk phenotypes; anti-inflammatory trials needed
Systemic inflammatory response	Ischemia-reperfusion injury, hypoperfusion, and immune activation trigger exaggerated cytokine release	SIRS criteria, hs-CRP, IL-6, WBC, NLR, NPAR	Acute kidney injury, prolonged ICU stay, ↑ mortality	Serial biomarker monitoring; early recognition of hyperinflammatory trajectories
Thromboinflammatory leaflet injury	Blood–prosthesis interaction promotes fibrinogen/vWF deposition, platelet activation, and leukocyte recruitment	Platelet dynamics, inflammatory biomarkers; CT: HALT/HAM	Subclinical leaflet thrombosis, impaired leaflet motion	CT surveillance; antithrombotic/anti-inflammatory modulation trials
Structural valve degeneration	Persistent thromboinflammation, fibrosis, xenoantigen-driven immunity, and calcification	Histopathology; CT leaflet thickening; serial echo gradients	Structural valve deterioration, late prosthetic dysfunction, HF recurrence	Long-term biomarker/imaging follow-up; novel valve coatings (preclinical)
Baseline inflammatory burden	Inflammaging, comorbidities (CKD, DM, COPD, obesity) lower threshold for hyperactivation	hs-CRP, IFN-γ, WBC, BNP, creatinine, albumin; multimarker panels	↑ Mortality, rehospitalization, adverse trajectories	Pre-procedural inflammatory phenotyping; comorbidity optimization
Gut microbiota-immune axis	TMAO promotes valvular interstitial cell fibrotic activation and macrophage M1 polarization via ER stress and m6A pathways	TMAO (exploratory)	Possible amplification of post-procedural inflammatory burden	Exploratory; microbiome-based strategies under investigation
Epicardial adipose tissue inflammation	Inflamed EAT acts as paracrine source of cytokines and fibrosis-promoting signals	CT-derived epicardial adipose tissue attenuation	↑ MACCE, Heart failure-related readmissions	Pre-TAVI CT enriches prognostic assessment without additional invasiveness

Abbreviations: BNP, B-type natriuretic peptide; CKD, chronic kidney disease; COPD, chronic obstructive pulmonary disease; CRP, C-reactive protein; CT, computed tomography; DAMPs, damage-associated molecular patterns; DM, diabetes mellitus; echo, echocardiography; ER, endoplasmic reticulum; HALT, hypoattenuated leaflet thickening; HAM, hypoattenuated leaflet motion; hs-CRP, high-sensitivity C-reactive protein; ICU, intensive care unit; IFN-γ, interferon-gamma; IL-6, interleukin-6; m6A, N6-methyladenosine; MACCE, major adverse cardiovascular and cerebrovascular events; NLR, neutrophil-to-lymphocyte ratio; NPAR, neutrophil percentage-to-albumin ratio; PPI, permanent pacemaker implantation; SIRS, systemic inflammatory response syndrome; TMAO, trimethylamine N-oxide; vWF, von Willebrand factor; WBC, white blood cell count; EAT, epicardial adipose tissue; HF, heart failure. ↑ means “higher’’.

## 10. Therapeutic Modulation of Inflammation: Lessons From Ischemic Heart Disease and Implications for TAVI

Targeting the NLRP3–IL-1β–IL-6 axis has been extensively investigated in ischemic heart disease, providing proof-of-concept that inflammation is a modifiable therapeutic target in cardiovascular disease [6,7,89,90]. Although the pathophysiological context differs from TAVI, peri-procedural sterile inflammation after valve implantation converges on many of the same inflammatory pathways. Therefore, evidence derived from ischemic heart disease may offer a translational framework for the design of future biomarker-guided anti-inflammatory strategies in selected TAVI populations.

In the CANTOS trial [9], canakinumab reduced hs-CRP and IL-6 and lowered recurrent cardiovascular events, while colchicine showed benefits in chronic coronary artery disease and post-myocardial infarction (LoDoCo2 [79] and COLCOT [78]). However, neutral findings in CLEAR [91] and COLICA [92] trials highlight the importance of clinical setting, timing, and patient selection. Overall, these studies established inflammation as a relevant therapeutic target in cardiovascular disease rather than a passive correlate of injury.

Building on this concept, IL-1 blockade with anakinra has demonstrated the ability to reduce systemic inflammation and the composite risk of death and heart failure following ST-segment elevation myocardial infarction (STEMI) [93,94,95,96], and goflikicept (RPH-104), a novel IL-1α/β trapping fusion protein, has shown significant anti-inflammatory efficacy and promising clinical signals in STEMI patients [97]. The ongoing VA-ART4 trial (Interleukin-1 Blockade in Acute Myocardial Infarction to Prevent Heart Failure; NCT05177822) is testing whether very early IL-1 blockade can prevent adverse ventricular remodeling and new-onset heart failure after STEMI [98].

IL-6 inhibition is also being explored: tocilizumab reduced post-myocardial infarction (MI) microvascular obstruction but did not improve infarct size or short-term MACE in ASSAIL-MI [99], while ziltivekimab showed strong anti-inflammatory effects in the RESCUE trial [100]. Several phase 3 trials are currently evaluating ziltivekimab in atherosclerotic cardiovascular disease (ZEUS, NCT05021835) [101], heart failure with preserved or mildly reduced ejection fraction (HERMES [102]; ATHENA, NCT06200207), and post-myocardial infarction (ARTEMIS, NCT06118281).

Upstream targeting of the NLRP3 inflammasome is another promising approach. The selective NLRP3 inhibitor MCC950 demonstrated potent anti-inflammatory effects in human carotid atherosclerotic plaques, markedly reducing interleukin-1β levels and macrophage proliferation by more than 47% in *ex vivo* plaque cultures [103]. Notably, this antiproliferative effect on inflammatory cells within atherosclerotic plaques appeared independent of IL-1β supplementation, suggesting broader mechanisms of action within the inflammatory cascade [103].

Dapansutrile (OLT1177), an oral NLRP3 inhibitor, reduced hs-CRP with a favorable safety profile in early inflammatory disease studies, although cardiovascular outcomes remain unknown [7,104,105]. Inclacumab, an anti–P-selectin antibody, reduced biomarkers of periprocedural myocardial injury in non-ST-segment elevation myocardial infarction (NSTEMI) patients undergoing percutaneous coronary intervention (PCI) (SELECT-ACS trial [106]), but long-term outcomes are pending. Ongoing trials continue to explore anti-inflammatory strategies in ischemic heart disease, driven by the impact of residual inflammatory risk on MACE and mortality [67]. COLCARDIO-ACS (ACTRN12616000400460) and COL-BE PCI (NCT06095765) are assessing colchicine in acute coronary syndrome (ACS) and post-PCI settings, while DOBERMANN-T (NCT05350592) evaluates tocilizumab plus low-dose dobutamine in high-risk STEMI patients. Beyond the IL-1/IL-6 pathway, RITA-MI2 (rituximab, anti-CD20, in STEMI) [107] showed safety and B-cell depletion, whereas GOLDILOX-TIMI 69 (anti-lectin-like oxidized low-density lipoprotein receptor-1 (LOX-1)) improved inflammatory biomarkers but did not reduce noncalcified plaque volume after MI [108].

Of note, several anti-inflammatory strategies failed to improve cardiovascular outcomes, highlighting the need for appropriate pathway targeting and patient selection. Methotrexate (CIRT trial) showed no reduction in cardiovascular events or inflammatory biomarkers in patients with stable atherosclerosis [109]. Darapladib, a lipoprotein-associated phospholipase A2 (Lp-PLA2) inhibitor, failed to reduce major adverse cardiovascular events in both the STABILITY and SOLID-TIMI 52 trials despite reducing Lp-PLA2 activity [110]. Losmapimod, a p38 mitogen-activated protein kinase (MAPK) inhibitor, showed no benefit in the LATITUDE-TIMI 60 trial [111]. Varespladib, a secretory phospholipase A2 (sPLA2) inhibitor, failed in the VISTA-16 trial, likely due to nonspecific inhibition of both pro-atherogenic and atheroprotective sPLA2 isoforms [112]. Pexelizumab, a complement C5 inhibitor, also failed to demonstrate cardiovascular benefit [113]. Collectively, the failure of these agents—despite targeting diverse inflammatory pathways—underscores that anti-inflammatory efficacy in cardiovascular disease depends critically on targeting the correct pathway, in the right clinical context, with appropriate patient selection. These negative and neutral findings are not peripheral to the narrative of anti-inflammatory therapy in cardiovascular disease—they are central to it. The failure of broad-spectrum or pathway-nonspecific anti-inflammatory agents highlights that not all inflammatory targets are equally relevant across clinical settings, and that efficacy depends on the precise alignment between the targeted pathway, the timing of intervention, and the patient population. In the context of TAVI, this principle is particularly important: the GLUCO-TAVI trial showed no benefit of corticosteroids on pacemaker implantation, and the Co-STAR trial—while providing proof-of-concept signals—was terminated early due to a safety concern, reinforcing that even biologically plausible strategies require rigorous TAVI-specific validation before clinical adoption. These insights are relevant to TAVI, where peri-procedural inflammation involves the NLRP3–IL-1β–IL-6 axis, but is also influenced by procedural trauma and patient characteristics as aforementioned. Accordingly, therapeutic strategies should focus on selective modulation of high-risk inflammatory profiles, supporting biomarker-guided trials in carefully selected TAVI populations (Fig. 2).

**Fig. 2.**
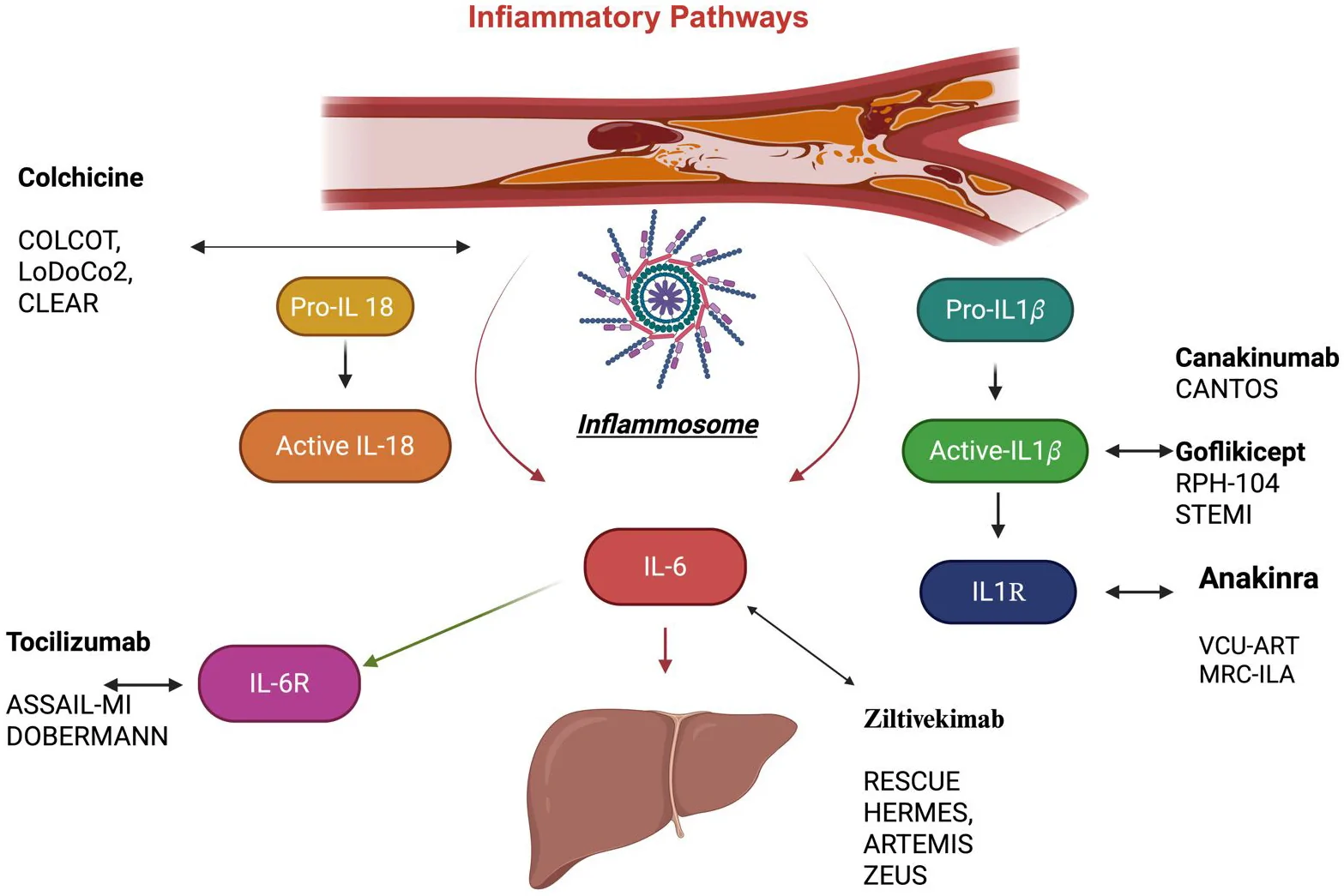
**Main inflammatory pathways and potential anti-inflammatory therapy with respective trials**. Sterile tissue injury activates the NLRP3 inflammasome, leading to cleavage of pro–interleukin (IL)-1β and pro–IL-18 into their biologically active forms. Active IL-1β amplifies innate immune signaling and promotes downstream IL-6 production, which drives the hepatic acute-phase response and systemic inflammation. Pharmacologic interventions can target different levels of this cascade. Upstream inhibition of inflammasome activation is achieved with colchicine, which disrupts inflammasome assembly and signaling (COLCOT, LoDoCo2, CLEAR). IL-1β signaling can be blocked directly with canakinumab (CANTOS) or neutralized through IL-1α/β trapping with goflikicept (RPH-104). Anakinra inhibits downstream IL-1 receptor (IL-1R) activation, preventing IL-1–mediated inflammatory signaling (VCU-ART, MRC-ILA). Further downstream, IL-6 signaling may be attenuated by receptor blockade with tocilizumab (ASSAIL-MI, DOBERMANN) or by direct IL-6 neutralization with ziltivekimab (RESCUE, HERMES, ARTEMIS, ZEUS). By targeting sequential steps of the NLRP3–IL-1β–IL-6 pathway, these therapies provide a translational framework for anti-inflammatory strategies that may be applicable to patients undergoing TAVI. Figure created with BioRender.

Importantly, direct extrapolation of findings from ischemic heart disease to the TAVI setting should be undertaken cautiously. Whereas inflammation following myocardial infarction primarily results from ischemic myocardial injury, peri-TAVI inflammation reflects a more complex interplay between procedural trauma, endothelial disruption, ischemia-reperfusion injury, biomaterial-related immune activation, and patient-specific susceptibility. These mechanistic differences may affect both the choice of therapeutic target and the timing of intervention, and underscore the need for TAVI-specific anti-inflammatory trials rather than reliance on extrapolated evidence.

## 11. Challenges and Unanswered Questions

Despite growing evidence, several key questions remain. First, the causal role of inflammation has not been definitively established: it remains unclear whether inflammatory biomarkers directly mediate adverse outcomes or primarily reflect procedural complexity and patient frailty. This distinction is critical, as it determines whether inflammation should be regarded as a prognostic marker or a true therapeutic target.

Second, the field lacks standardized criteria for defining pathologic hyperinflammation after TAVI. Current SIRS-based definitions are nonspecific and may inadequately capture the distinct inflammatory profile of this population, contributing to diagnostic uncertainty and, in some cases, unnecessary antibiotic use. More refined definitions integrating clinical features with serial biomarkers—such as hs-CRP, IL-6, leukocyte count, and NLR—are needed.

Third, the long-term consequences of persistent inflammation on valve durability remain incompletely understood. Chronic inflammatory activation may contribute to leaflet thrombosis, fibrosis, and structural valve deterioration—an issue of increasing relevance as TAVI expands to younger patients with longer life expectancy. Standardized biomarker sampling schedules and integration with multimodality imaging will be essential to clarify this relationship.

Finally, whether the inflammatory response to TAVI has implications for other transcatheter valve procedures, such as transcatheter edge-to-edge mitral repair—which may also induce inflammation associated with increased mortality—deserves further investigation.

## 12. Conclusions

Peri-procedural inflammation has emerged as a relevant determinant of clinical outcomes after TAVI that extends beyond a mere marker of procedural injury. The magnitude and consequences of this response vary considerably among patients, reflecting the complex interplay between baseline inflammatory susceptibility, procedural factors, and device-related characteristics. Incorporating inflammatory biomarkers, multimodality imaging, and individualized inflammatory subphenotyping into clinical assessment may enhance risk stratification and facilitate a more personalized approach to post-TAVI management. However, although inflammation represents an attractive candidate therapeutic target, robust evidence demonstrating that targeted anti-inflammatory interventions improve clinical outcomes in this setting is still lacking; accordingly, inflammation after TAVI should currently be regarded as a prognostic factor and an area of active investigation rather than a validated therapeutic target. Future research should focus on establishing standardized definitions of pathologic post-TAVI inflammation, validating biomarker- and imaging-guided risk stratification strategies, clarifying whether persistent inflammation contributes to long-term valve durability and structural valve degeneration, and testing targeted anti-inflammatory interventions in biomarker-selected high-risk populations. Addressing these priorities will be essential to determine whether peri-procedural inflammation can be transformed from a prognostic indicator into a modifiable therapeutic target in patients undergoing TAVI.
